# Systematic scoping review of factors and measures of rurality: toward the development of a rurality index for health care research in Japan

**DOI:** 10.1186/s12913-020-06003-w

**Published:** 2021-01-04

**Authors:** Makoto Kaneko, Ryuichi Ohta, Evelyn Vingilis, Maria Mathews, Thomas Robert Freeman

**Affiliations:** 1 Primary Care Research Unit, Graduate School of Health Data Science, Yokohama City University, 22-2, Seto, Kanazawa-ku, Yokohama, Kanagawa 236-0027 Japan; 2 Department of Family and Community Medicine, Hamamatsu University School of Medicine, 1-20-1, Handayama, Higashi-ku, Hamamatsu, 431-3192 Japan; 3 Department of Family Medicine, Schulich School of Medicine & Dentistry, Western Centre for Public Health and Family Medicine, London, Ontario Canada; 4 Department of Community Care, Unnan City Hospital, 699-1221 96-1 Iida, Daito-Cho, Unnan City, Shimane Japan

**Keywords:** Geography, Health services research, Japan, Rurality index, Scoping review

## Abstract

**Background:**

Rural-urban health care disparities are an important topic in health services research. Hence, developing valid and reliable tools to measure rurality is needed to support high quality research. However, Japan, has no index to measure rurality for health care research. In this study, we conducted a systematic scoping review to identify the important factors and methodological approaches to consider in a rurality index to inform the development of a rurality index for Japan.

**Methods:**

For our review, we searched six bibliographic databases (MEDLINE, PubMed, CINAHIL, ERIC, Web of Science and the Grey Literature Report) and official websites of national governments such as Government and Legislative Libraries Online Publications Portal (GALLOP), from 1 January 1989 to 31 December 2018. We extracted relevant variables used in the development of rurality indices, the formulas used to calculate indices, and any measures for reliability and validity of these indices.

**Results:**

We identified 17 rurality indices from 7 countries. These indices were primarily developed to assess access to health care or to determine eligibility for incentives for health care providers. Frequently used factors in these indices included population size/density and travel distance/time to emergency care or referral centre. Many indices did not report reliability or validity measures.

**Conclusions:**

While the concept of rurality and concerns about barriers to access to care for rural residents is shared by many countries, the operationalization of rurality is highly context-specific, with few universal measures or approaches to constructing a rurality index. The results will be helpful in the development of a rurality index in Japan and in other countries.

## Background

Addressing rural-urban health care disparities is an important health system challenge. Numerous studies have reported that rural residents are more likely to have chronic diseases related to obesity, and less likely to engage in healthy behaviours, compared to urban residents [1–4]. Rural residents have poorer access to health care providers and have fewer visits to family physicians and specialists than urban residents [5, 6]. Moreover, living in a rural area is associated with lower physical/social functioning, mental health, self-reported health status [7], cancer survival [8], and overall quality of life [9]. Also, the recruitment and retention of the health care providers are major challenges in rural areas [10]. For example, Ontario, Canada originally developed the Rurality Index of Ontario (RIO) [11] in 2000 for policy purposes such as workforce incentives targeting physician recruitment and retention in rural areas [11]. Australia’s Modified Monash Model (MMM) [12] was based on Humphreys’s (2012) paper [13], and has been used to develop the recruitment and retention programs for health care providers [12]. However, a challenge for the development of rural indices is that many definitions of rurality exist as “rural” areas can include a wide range of community characteristics (e.g. level of affluence, degree of industrialization) [14, 15]. Yet most definitions of rurality focus on geographic characteristics (e.g., low population density or distance from health care resources) [14, 15], and not on social/cultural issues such as “way of life”, and “state of mind” associated with rural living that can affect health disparities [15]. Additionally, different methods have been used to calculate these indices. For example, the RIO used a sum of community population, travel time to nearest referral centre and travel time to nearest advanced referral centre to produce a continuous variable from 0 to 100 [11] while the MMM used a combination of population size and geographical remoteness to provide a 7-level classification with 1 representing a major city and 7 representing a high level of remoteness [12].

In Japan, researchers and policy makers do not have a rurality index. Japan has 6800 islands and 683,000 (0.5% of overall population) live on these islands [16]. Also, 11 million people live in rural areas called “depopulated areas” (11% of overall and the area is 58% of all areas) and 130,000 people live in “districts without a doctor (the areas are defined as the area over 50 residents within a radius of 4 km with limited access) [16]. Although the national government classified the “depopulated areas” based on the municipality’s income, demand and population trends, it is determined subjectively with no concrete definition or formula to consistently apply [17]. This existing index fails to capture the variation in health status and physician resources, ignores socio-cultural considerations, and is not widely accepted by primary care physicians [18].

In order to report comparisons between communities, researchers from various jurisdictions have developed indices to measure and categorize different degrees of rurality. Ensuring that a rurality index is valid and reliable is an integral step in promoting the widespread acceptance of the index, gathering high quality data, and developing strategies to tackle health and health workforce disparities [19].

The goal of this study is to identify the important factors and methods of measuring rurality for health services research and health policy. This scoping review is the first step in a project to develop a rurality index for Japan. The findings will also be useful for other countries and rural health researchers.

### Aims

The aims of the scoping review are to 1) describe the publication characteristics of rurality indices, 2) identify factors commonly used in rurality indices, and 3) assess validity and reliability properties of published rurality indices.

## Methods

### Study design

A systematic scoping review.

A systematic scoping review is a review of existing literature to clarify a complex concept and refine subsequent research [20]. Usually, a systematic scoping review does not assess the quality of included studies, unlike a systematic review [20]. Also, a systematic scoping review is different from a narrative review because the scoping process requires analytical reinterpretation of the included literature [20]. A systematic scoping review is suitable for a discipline in which the shortage of randomized control trials makes it difficult for investigators to conduct a systematic review [20].

Arksey and O′Malley [21] presented a five-stage methodological framework to be used for scoping reviews that was further expanded by Levac et al. [20] The framework includes the following stages: identification of the research question; identification of relevant studies; selection of studies; charting of the data; collation, summarizing and reporting the results.

The systematic scoping review answers the research question: what are the factors, methods, and any measures for reliability and validity of rurality indices used in health care and health system related research.

### Search strategy (identifying relevant studies)

We included articles and websites in English and Japanese. We searched the following bibliographic databases (MEDLINE, PubMed, CINAHIL, ERIC, Web of Science and the Grey Literature Report) to identify relevant papers. Also, we searched Government and Legislative Libraries Online Publications Portal (GALLOP), Registry of Canadian Government Information Digitization Projects, Canadian Research Index – Microlog, Municipal Information Network, Canadian Public Policy Collection, United Nations digital library, the US Census website and Organization for Economic Co-operation and Development (OECD) library to look identify rurality indices employed by national/local governments around the world. Moreover, we used Japanese databases, Ichushi-Web, an online Japanese literature searching system provided by the non-profit Japan Medical Abstracts Society. Ichushi-Web includes roughly 10 million medical papers from 6000 journals in Japan and is often used for Japanese literature searches [22]. We included studies that were published from 1 January 1989 to 31 December 2018 (the last 30 years) and websites. The most recent search date was 10th September 2019. The search strategy was based on the following title/abstract keywords in English and Japanese: “rurality” OR “rurality index” OR “index of rurality” OR “rurality measurement” OR “remoteness index” OR “accessibility index” OR “population density index”. Moreover, we added “develop” OR “create” OR “construct” OR “generate”. In addition, we used MESH term: “Rural Health Services/classification” OR “peripherality index”. We also reviewed the reference lists of relevant studies to identify research that might have been missed in the database search.

### Inclusion/exclusion criteria (study selection)

Literature searches and study selection were independently conducted by two investigators (MK and RO) and any discrepancies were resolved by discussion.

We excluded articles and websites that used a previously developed rurality index.

### Data extraction (charting data)

To report each study, we followed the approach of described in the PRISMA Extension for Scoping Reviews (PRISMA-ScR): Checklist and Explanation [23]. This checklist includes 22 essential reporting items that reflect on the title, abstract, introduction, methods, results, discussion and funding [23]. Based on an initial search of the literature, we developed an extraction template that included the following elements:

title of the article, name of authors, years of publication, name of the journal/website, name of the index, publication status (yes/no), peer review status (yes/no), citation index by Web of Science, country/province, unit of analysis (geographical jurisdiction/health care institution/individual), types of variable of rurality index (continuous/categorical), purpose of the index (for general purpose/for health care policy and research), study design, selection of factors included in the index, reported measures of reliability and validity.

We classified the unit of analysis into three categories: geographical jurisdiction, health care institution, or individual. The unit of analysis may vary depending on the purpose of the index. To assess rurality in specific areas, geographical jurisdiction is suitable for unit of analysis. Health care institution is employed for measurement of rurality from the viewpoint of each medical institution. Individual-level rurality is used for assessing each person’s accessibility to health care.

We categorize the purpose of the index into two categories: for general purpose and for health care policy and research. An index created for general purposes can be used for various situations. An index created for health care policy or research purposes is developed to measure rurality specific to health care.

## Results

**Fig. 1:**
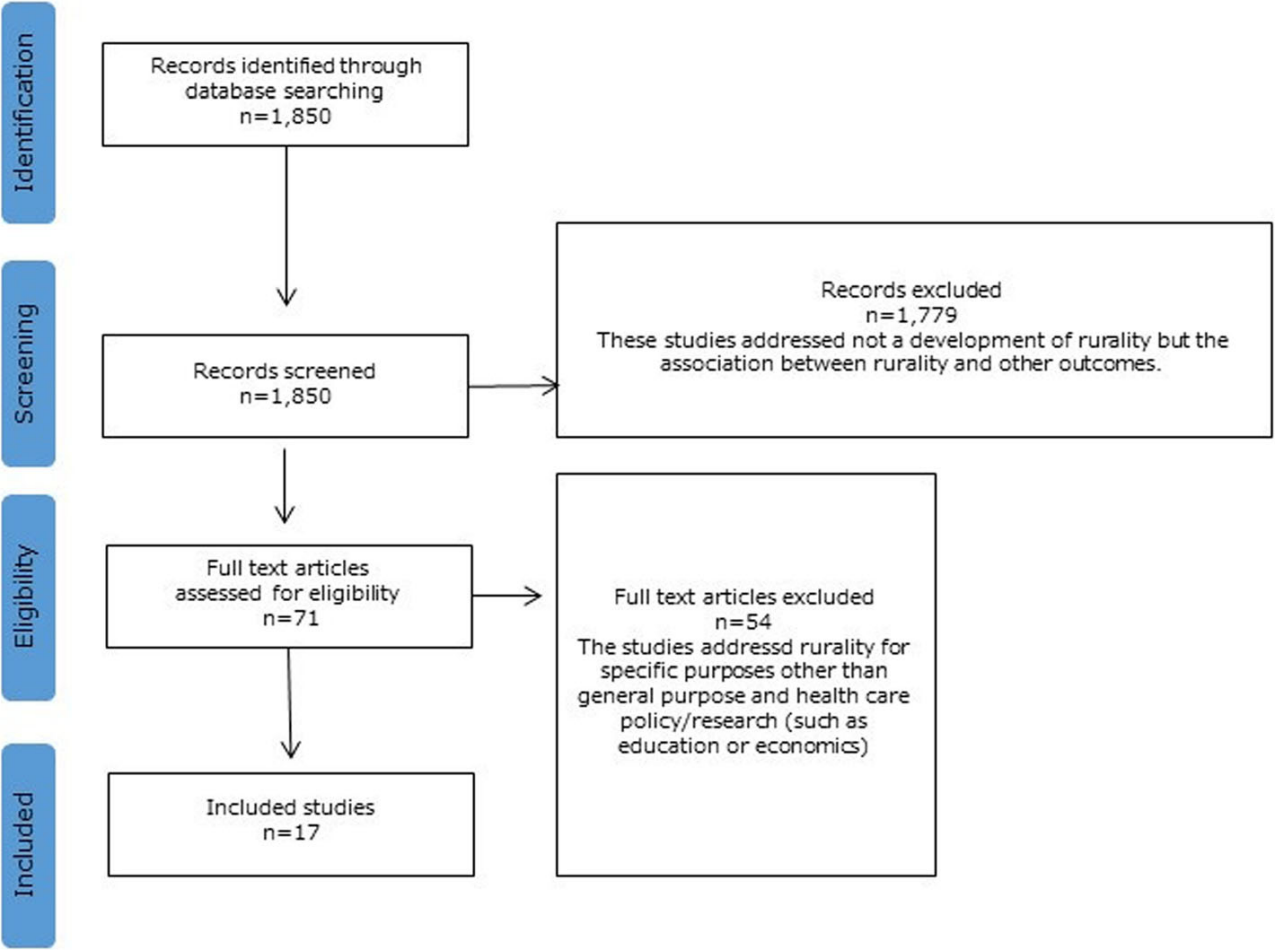
Flow diagram for the selection of studies in the systematic scoping review

After searching through the titles and abstracts of 1850 publications, 17 eligible publications [11, 12, 24–38] were identified. Reasons for exclusion are shown in Fig. 1.

### Publication characteristics of the rurality indices

As shown in Table 1, 14 (82%) of the 17 articles have been published since 2000. Table 2 shows that the majority (13; 76%) have been published in Australia, Canada and the US. Three indices were developed in Europe (Germany, Italy, and Scotland) and only one index was developed in Asia (China). Of 17 indices, 12 (71%) were published in a peer-reviewed journal and five were posted online on the web.

**Table 1:** Publication characteristics of the rurality indices

Author and publication year	Country	Peer review (yes/no)	Citation index by Web of Science
Department of Primary Industries and Energy, Department of Human Services and Health, 1994 [24]	Australia	No	not included
Weinert et al., 1995 [25]	USA	Yes	38
Leduc, 1997 [26]	Canada	Yes	not included
Department of Health and Aged Care, 2001 [27]	Australia	No	not included
Australian Institute of Health and Welfare Canberra, 2004 [28]	Australia	No	not included
Swan et al., 2008 [29]	Scotland	Yes	9
Kralj, 2008 [11]	Canada	No	not included
McGrail et al., 2009 [30]	Australia	Yes	61
Han et al., 2012 [31]	China	Yes	9
Humphreys et al., 2012 [13]	Australia	Yes	not included
Steinhaeuser et al., 2014 [32]	Germany	Yes	8
Mao et al., 2015 [33]	USA	Yes	4
Zhu et al., 2015 [34]	USA	Yes	not included
Inagami et al., 2016 [35]	USA	Yes	3
Alasia et al., 2017 [36]	Canada	No	6
Calovi et al., 2018 [37]	Italy	Yes	0
Doogan et al., 2018 [38]	USA	Yes	0

**Table 2:** Description of purpose, types of factors included, and unit of analysis of rurality indices

Author and publication year	Name of the index	Purpose of the index (for general purpose or health care policy and research)	Types of variables: continuous or categorical	Unit of analysis	How to decide the included factors
Department of Primary Industries and Energy, Department of Human Services and Health, 1994 [24]	Rural, remote and metropolitan area (RRMA)	for general purpose	categorical	geographical jurisdiction: Statistical Local Area	consensus of a working group
Weinert et al., 1995 [25]	MSU rurality index	for health care policy and research	continuous	individual	literature review and availability of the data
Leduc, 1997 [26]	General Practice Rurality Index (GPRI)	for health care policy and research	continuous	health care institution: general practice	literature review
Department of Health and Aged Care, 2001 [27]	Accessibility/Remoteness Index of Australia (ARIA)	for general purpose	continuous	geographical jurisdiction: populated location	GIS network analysis
Australian Institute of Health and Welfare Canberra, 2004 [28]	Australian Standard Geographical Classification (ASGC)	for general purpose	continuous	geographical jurisdiction: Statistical Local Area	An enhanced measure of previous remoteness index, ARIA+
Swan et al., 2008 [29]	Clinical peripherality indicator	for health care policy and research	continuous	health care institution: general practice	factor analysis
Kralj, 2008 [11]	Rurality Index for Ontario (RIO)	for health care policy and research	continuous	geographical jurisdiction: community	principal component analysis, maximum likelihood method
McGrail et al., 2009 [30]	Index of Rural Access	for health care policy and research	continuous	geographical jurisdiction: collection district	modified two-step floating catchment area method
Han et al., 2012 [31]	Rural PHCWA index	for health care policy and research	continuous	geographical jurisdiction: county	literature review
Humphreys et al., 2012 [13]	six-level geographical classification	for health care policy and research	categorical	geographical jurisdiction: city	Geo-coded data and the previous study
Steinhaeuser et al., 2014 [32]	modified RRS-Germany (mRRS-G)	for health care policy and research	continuous	health care institution: general practice	translation and adaptation of the previous rurality index, RRS
Mao et al., 2015 [33]	Individual-based rurality and well-being measures	for health care policy and research	continuous	individual	active space approach
Zhu et al., 2015 [34]	Rural taxonomy	for health care policy and research	categorical	geographical jurisdiction: primary care service area	cluster analysis
Inagami et al., 2016 [35]	IRR zip	for health care policy and research	continuous	geographical jurisdiction: zip-code level	modification of the previous rurality index, Index of Relative Rurality
Alasia et al., 2017 [36]	index of remoteness	for general purpose	continuous	geographical jurisdiction: census subdivision	gravity model
Calovi et al., 2018 [37]	spatial accessibility index	for health care policy and research	continuous	geographical jurisdiction: municipality	two-step floating catchment area method
Doogan et al., 2018 [38]	Isolation scale	for health care policy and research	continuous	geographical jurisdiction: census tract	literature review

*MSU* Montana State University, *GIS* Geographic Information System, *PHCWA* Primary Health Care Worker Accessibility index, *RRS* Rural Ranking Scale, *IRR* Index of Relative Rurality

### Purposes of and factors in rurality indices

Thirteen indices (76%) were developed for health care policy or research purposes and four (24%) were developed for general purpose (Table 2). The indices developed for health care policy or research were primarily designed to measure access to health care resources or to determine incentives for health care providers based on rurality. Fourteen indices (82%) measured rurality as a continuous score.

The unit of analysis in each study was determined by the purpose of the index (Table 2). Twelve indices (71%) employed geographical jurisdiction (such as statistical local area, county, state, or postal code) as a unit of analysis. Three focused on a medical institution (general practice) and two targeted individuals as the unit of analysis. The factors included in each rurality index are shown in Table 3. These factors were included in an index based on previous literature search and the availability of data related to the factor. The most frequently used factor was population (size or density) (*n* = 11: 65%). Travel distance and time to emergency care and/or referral centre were also often employed (*n* = 7: 41% and 3: 18%, respectively). In four indices (24%), resource availability expressed either as the number of physicians (both primary care and specialists) or as physician/population ratio was included in the index.

**Table 3:** Publication details and included factors of rurality indices

Author and publication year	Name of the index	Population yes/no	Travel distance yes/no	Travel time yes/no	Travel cost yes/no
Department of Primary Industries and Energy, Department of Human Services and Health, 1994 [24]	Rural, remote and metropolitan area (RRMA)	yes (population size)	no	no	no
Weinert et al., 1995 [25]	MSU rurality index	yes (population size)	yes (distance to emergency care)	no	no
Leduc, 1997 [26]	General Practice Rurality Index (GPRI)	yes (population size)	yes (distance to basic/advanced referral center)	no	no
Department of Health and Aged Care, 2001 [27]	Accessibility/Remoteness Index of Australia (ARIA)	no	yes (distance to the nearest centre)	no	no
Australian Institute of Health and Welfare Canberra, 2004 [28]	Australian Standard Geographical Classification (ASGC)	no	yes (distance to the nearest centre/the service town)	no	no
Swan et al., 2008 [29]	Clinical peripherality indicator	yes (population density)	no	yes (travel time to nearest specialist led hospital and to Health Board administrative headquarters)	no
Kralj, 2008 [11]	Rurality Index for Ontario (RIO)	yes (population size and population density)	no	yes (travel time to nearest basic referral centre and to nearest advanced referral centre)	no
McGrail et al., 2009 [30]	Index of Rural Access	yes: (population size)	no	no	no
Han et al., 2012 [31]	Rural PHCWA index	yes (population density)	no	no	no
Humphreys et al., 2012 [13]	six-level geographical classification	yes (population size)	yes (geographical remoteness)	no	no
Steinhaeuser et al., 2014 [32]	modified RRS-Germany (mRRS-G)	no	no	yes (travel time from the practice to next major hospital, to the nearest general practitioner colleague at place of work, to the satellite clinic and to most distant boundary covered by the practice)	no
Mao et al., 2015 [33]	Individual-based rurality and well-being measures	yes (population density)	no	no	no
Zhu et al., 2015 [34]	Rural taxonomy	no	no	no	no
Inagami et al., 2016 [35]	IRR zip	yes (population size and density)	yes (distance to metropolitan statistical area/micropolitan statistical area)	no	no
Alasia et al., 2017 [36]	index of remoteness	yes (population size)	no	yes (travel time)	yes
Calovi et al., 2018 [37]	spatial accessibility index	no	yes (distance to outpatient clinics)	no	no
Doogan et al., 2018 [38]	Isolation scale	no	yes	yes	no
Health care resources yes/no	Health care needs yes/no	Others	Formula		
no	no	level in urban hierarchy (small/large/metropolitan/capital city urban center)	not applicable		
no	no		Four mathematical operations are performed as below: 1. Distance and population measures are transformed to make the distribution of the resulting index as normal as possible 2. The transformed distance and population measures are standardized so that each has a standard deviation of one 3. The standardized transformed distance and population measures are weighted to produce an initial index of rurality that assigns high scores to rural families and low scores to urban families 4. The initial index constructed in operation #3 is restandardized to have a mean of zero and a standard deviation of one		
yes (number of general practitioners, number of specialists, presence of an acute care hospital)	no		Sum the points for each of the following (maximum 100 points): 1. Remoteness from closest advanced referral centre (km) ÷ 50 2. Remoteness from closest basic referral centre (km)÷25 3. 20*(Drawing population÷2000) 4. (20 ÷ number of full-time GPs with main place of business within 25 km of the centre of the community 5. Number of specialists 6. Presence of an acute care hospital		
no	no		unweighted addition of the four (threshold-limited) ratio values for each of the four levels of service centre		
no	no		calculates distance to the nearest centre in each of five categories of service centre		
no	yes (number of patients on the practice list)		Practice list size, ward population density and travel time to hospital were log transformed to achieve near normality. The relationships among the variables were assessed by matrix plots and correlation coefficients. This was further multiplied by 100 for the index to range from 0 to 100 with a midpoint of 50. Higher values represent greater peripherality.		
no	no		Sum the points for each of the following (maximum 100 points): 1. Measure of community population and population density 2. Measure of travel time to nearest basic referral centre 3. Measure of travel time to nearest advanced referral centre		
yes (the number of full-time equivalent services at location and the population-to-provider ratio)	yes (health needs (Disability Adjusted Life Years: DALYs))	mobility (households without a car, individuals of low personal mobility and public transport availability)	${\sum}_j^{\left\{\mathrm{100,10}\mathit{\min}\right\}}f2(dij)$*Rj*Mob*i* f(dij): impedance function R*j*: the population-to-provider ratio for service j Mob*i*: equal to one within the initial catchment (10 min), and is less than one in the secondary catchment for areas of low mobility		
yes (primary health care worker density per 1000 farming population index)	no		Rural PHCWA index of X province = primary health care worker density per 1000 farming population index of X province * population density index of X province.		
no	no		not applicable		
yes (backup by a paramedic team within 15 min and numbers of GP which engaged in on-call duty)	no		Sum the following six variables: 1. travelling time from the surgery to major hospital 2. on-call duty 3. receiving timely backup by a paramedic team 4. travelling time to nearest general practitioner colleague at place of work 5. travelling time to most distant practice boundary 6. satellite clinic		
yes (density of health facilities/social service facilities)	no	number of different ethnic groups/degree of land development/mean household income/density of loads	$\sum \limits_{L=1}^n ProbL,i$*RuralDegree_*L/*_ $\sum \limits_{L=1}^n ProbL,i$ 1. n is the total number of places within individual *i*’s activity space 2*. L* represents any one of these places 3. Prob*L,i* is the probability of visiting place *L* by individual *i* 4. the degree of rurality for all places (RuralDegreeL) were extracted with GIS database		
yes (provider resources: primary care physicians, medical specialists, non-physician practitioners, dentists and facility resources: staffed hospital beds, provider resources, average daily census, Medicare/Medicaid certified nursing home beds)	no	economic resource, age distribution	not applicable		
no	no		Step 1: Calculating maximum, minimum and range of each variable. Step 2: transforming each variable so that it is measured on a scale from 0 to 1. Step 3: calculating averages of the transformed variables The included variables are below: 1. population size, 2. population density 3. distance to closest metropolitan area		
no	no		ln $\sum \limits_{k=1}^n\left(\frac{Popk}{Ci,k}\right)$ Pop: sizes of the population centres C: travel cost		
no	no	volumes of activity	$$ \sum \limits_{\begin{array}{c}j\in \left\{ dij\leqq d0\right\}\\ {}\end{array}} Rj $$ d_*ij*_: the distance between *i* and *j* R*j*: supply-to-demand ratio at supply location j		
no	no		*v(i,j) = a*_*j*_*δ*^*dij*^ *a*_*i*_ = max_*j*_[*v*(*i,j*)] *v*: function *a*_*j*_: neighbor’s resources *d*_*ij*_: distance δ: parameter which chosen based on research purpose		

*MSU* Montana State University, *GIS* Geographic Information System, *PHCWA* Primary Health Care Worker Accessibility index, *RRS* Rural Ranking Scale, *IRR* Index of Relative Rurality

The 14 indices (82%) that calculated a continuous rurality score used a mathematical formula: e.g. summing up the included variables, log transformation or a more complex operation (Table 4). The formulas measured rurality as a continuous variable, such as 0 to 1 or 0 to 100. Geographic Information System (GIS) was used in five studies (29%), of which two studies (12%) employed a two-step floating catchment area method to assess geographical accessibility.

**Table 4:** Validity and reliability properties of rurality measures

Author and publication year	Name of the index	Reliability measures	Reliability score	Validity measures	Validity score
Department of Primary Industries and Energy, Department of Human Services and Health, 1994 [24]	Rural, remote and metropolitan area (RRMA)	not applicable	not applicable	not applicable	not applicable
Weinert et al., 1995 [25]	MSU rurality index	test/re-test	0.94 or larger	concurrent validity (comparison with other measure such as the participants’ perception)	*R*^2^ = 0.41, *r* = 0.85 and Z = 4.09
Leduc, 1997 [26]	General Practice Rurality Index (GPRI)	not applicable	not applicable	not applicable	not applicable
Department of Health and Aged Care, 2001 [27]	Accessibility/Remoteness Index of Australia (ARIA)	not applicable	not applicable	not applicable	not applicable
Australian Institute of Health and Welfare Canberra, 2004 [28]	Australian Standard Geographical Classification (ASGC)	not applicable	not applicable	not applicable	not applicable
Swan et al., 2008 [29]	Clinical peripherality indicator	not applicable	not applicable	not applicable	not applicable
Kralj, 2008 [11]	The Rurality Index for Ontario (RIO)	not applicable	not applicable	not applicable	not applicable
McGrail et al., 2009 [30]	the Index of Rural Access	not applicable	not applicable	not applicable	not applicable
Han et al., 2012 [31]	Rural PHCWA index	not applicable	not applicable	not applicable	not applicable
Humphreys et al., 2012 [13]	six-level geographical classification	not applicable	not applicable	concurrent validity	
Steinhaeuser et al., 2014 [32]	modified RRS-Germany (mRRS-G)	Cronbach’s alpha	negative	convergent construct validity	factor analysis: *R*^2^ = 59.4%
Mao et al., 2015 [33]	Individual-based rurality and well-being measures	not applicable	not applicable	not applicable	not applicabl
Zhu et al., 2015 [34]	Rural taxonomy	not applicable	not applicable	not applicable	not applicable
Inagami et al., 2016 [35]	IRR zip	not applicable	not applicable	face validity	not applicable
Alasia et al., 2017 [36]	the index of remoteness	not applicable	not applicable	not applicable	not applicable
Calovi et al., 2018 [37]	The spatial accessibility index	not applicable	not applicable	not applicable	not applicable
Doogan et al., 2018 [38]	Isolation scale	not applicable	not applicable	Spearman correlation to test convergent validity and the Akaike information criterion for criterion validity	Spearman correlation for convergent validity *r* = 0.99

*MSU* Montana State University, *GIS* Geographic Information System, *PHCWA* Primary Health Care Worker Accessibility index, *RRS* Rural Ranking Scale, *IRR* Index of Relative Rurality

### Validity and reliability properties

Fifteen indices (88%) did not examine reliability and 12 indices (71%) did not examine validity (Table 4). In some studies, test/re-test and Cronbach’s alpha were used to assess reliability. Validity was confirmed by examining correlation of the index with other measures.

## Discussion

The scoping systematic review found 17 rurality indices from seven countries. We found that these indices were designed specifically for health care research and policy purposes than general use. This review found that while the concept of rurality and concerns about barriers to access to care for rural residents is shared by many countries the approach to constructing an index was highly context specific. Although many indices were included, population size/density and travel time/distance to an advanced medical centre, none of the factors were used in all indices. These findings are consistent with earlier literature reviews that also found that a rurality index is generally based on population size or density and measures of distance such as travel time [39].

Although social, cultural, economic factors are associated with rurality [14, 15], none of the indices incorporated these factors. These factors may be relevant in the development of a rurality index for Japan. For example, the clinical peripherality index in Scotland accounted for local characteristics such as location on an island [29]. Similarly, Japan has many remote islands [16] and sometimes a patient can access a secondary hospital only by a ship or an airplane. The rurality index in Japan has to consider frequency/number of a round-trip flights or water transport. Additional travel related factors such as the quality of roads, availability of public transport, difficulty of the terrain, and weather (e.g. amount of annual snowfall) may be important considerations in the Japanese context.

In terms of reliability and validity, only 12 and 29% of all indices examined these measures, respectively. Reliability refers to the consistent interpretation and application. In terms of validity, content validity, such as face validity, may be more important than other forms of validity due to the highly contextual nature of the index [40]. Thus, gathering advice from health care providers and policy makers may be an important step in developing a rurality index for Japan.

### Study strengths

To the best of our knowledge, this is the first systematic scoping review about the methods and measures used in the development of a rurality index. Given the variability in the definition of rural and uses of a rurality index, a systematic review may never be an appropriate review method. However, summarizing information from existing indices through a scoping review s helpful in the development of new rurality index.

### Study limitations

This study has several limitations. First, we may have excluded potential indices, such as, an index developed for a specific research question or analysis that may not be generalizable or useful in developing a standardized rurality index. We also excluded classification schemes based on population or census area because these classifications do not take access to health care resources into account.

## Conclusion

We identified 17 rurality indices by conducting the systematic scoping review. Although the operationalization of rurality is highly context specific, some variables were frequently employed in multiple countries/areas. The results will be helpful to develop a rurality index in Japan and other countries/areas.

## Data Availability

All data relevant to the study are included in the article.

## Abbreviations

GALLOP: Government and Legislative Libraries Online Publications Portal
MMM: Modified Monash Model
OECD: Organization for Economic Co-operation and Development
RIO: Rurality Index of Ontario
